# CLDN6 promotes tumor progression through the YAP1-snail1 axis in gastric cancer

**DOI:** 10.1038/s41419-019-2168-y

**Published:** 2019-12-11

**Authors:** Site Yu, Yeqian Zhang, Qing Li, Zizhen Zhang, Gang Zhao, Jia Xu

**Affiliations:** 1 Department of General Surgery, Renji Hospital, School of Medicine, Shanghai Jiao Tong University, No. 160, Pujian Road, Shanghai, 200127 P.R. China; 2 State Key Laboratory of Oncogenes and Related Genes, Shanghai Cancer Institute, Renji Hospital, School of Medicine, Shanghai Jiaotong University, Shanghai, P.R. China

**Keywords:** Oncogenes, Epithelial-mesenchymal transition

## Abstract

Claudin6 (CLDN6), a member of the tight junction family, is a molecule involved in intercellular adhesion, acting as a physical barrier that prevents solutes and water from freely passing through the extracellular space. CLDN6 has important biological functions, and its abnormal expression is associated with Hepatitis C infection. However, there is limited research regarding its role in gastric cancer. In this study, we found that the expression of CLDN6 mRNA and protein was upregulated in gastric cancer cell lines and tissues, which indicated poor prognosis. Both in vitro and in vivo experiments showed that abnormal CLDN6 expression was associated with enhanced proliferation and invasion abilities of gastric cancer. CLDN6 reduced the phosphorylation of LATS1/2 and YAP1 by interacting with LATS1/2 in the Hippo signaling pathway. Thus, CLDN6 affected the entry of YAP1 into the nucleus, causing changes in downstream target genes. Moreover, YAP1 interacted with snail1 to affect the process of EMT and enhanced the invasive ability of GC cells. Collectively, CLDN6 promoted the proliferation and invasive ability of gastric cancer by affecting YAP1 and YAP1-snail1 axis.

## Introduction

Gastric cancer is one of the five major malignant tumors that seriously endanger human health^1^. The incidence of gastric cancer is higher in East Asian countries, especially in China, Japan, and South Korea^2^. In China, gastric cancer ranks second and third in annual morbidity and mortality, respectively^3^. While the rate of diagnosis of early gastric cancer has improved with the advancement of medical technology, the overall rate of diagnosis remains poor^4^. Most patients with gastric cancer are in the middle and late stage when diagnosed, and the malignant degree of these two stages is high, often causing distant visceral metastasis through blood and lymphatic drainage, and other mechanisms^5^. Studies have shown that in patients with advanced gastric cancer, the incidence of liver metastasis is as high as 44%, and the 5-year survival rate of patients is approximately 10%^6^. Therefore, it is of utmost importance to study the mechanism of proliferation and metastasis of gastric cancer to improve the therapeutic effect and prognosis^7,8^.

CLDN6 is a member of the claudin family and the membrane protein encoded by the gene is a component of tight junction. The molecular weight of claudin protein is approximately 20–40 kDa. The common characteristics of claudin proteins include a short intracytoplasmic N-terminal region, two extracellular ring domains formed by four transmembrane domains, and an intracytoplasmic C-terminal tail^9^. All claudin family members, except claudin12, have a carboxy-terminal PDZ binding motif that allows claudins to interact with cytoplasmic scaffolds^10^. The abnormal expression of CLDN6 is often associated with Hepatitis C infection^11,12^. However, there is sparse literature regarding its role in gastric cancer, and the mechanistic effect of claudin6 on tumorigenesis is still unclear. Epithelial mesenchymal transition (EMT) is an important process in tumorigenesis that is closely related to tumor invasion, metastasis, and prognosis. Recent studies have shown that EMT plays an important role in many biological processes such as malignant proliferation of tumor cells, regulation of tumor microenvironment, and activation of tumor stem cells^13–15^. In addition, a previous study reported the correlation between claudin proteins and EMT^16^. However, the role of CLDN6 in the malignant transformation of gastric cancer through the EMT network is still unknown.

In the present study, we found that CLDN6 was significantly upregulated in gastric cancer, and its abnormal increased expression often predicted poor prognosis of GC patients. CLDN6 interacted with LATS1/2, resulting in the reduction of YAP1 phosphorylation, thereby increasing YAP1 nuclear translocation and activating downstream target genes. Moreover, YAP1 interacted with snail1 and promoted the process of EMT. CLDN6 therefore, might be a novel prognostic molecule for gastric cancer and a potential target candidate for future treatment of gastric cancer.

## Materials and methods

### Patients and tissue samples

In this study, gastric cancer tissues were obtained from 494 patients who underwent surgical resection in Renji hospital from 2010 to 2016. After surgical resection, all samples were immediately frozen in liquid nitrogen and stored at −80 °C. The tissue samples were used to produce tissue microarrays for later use. The clinicopathological characteristics of the selected patients were obtained from their medical records. The guidelines published in the 7th edition of the TNM classification of tumors by the International Union Against Cancer (UICC) was used for histological classification of tumors according to the criteria set by the World Health Organization. This study was approved by the Ethics Committee of Renji Hospital and written informed consent was obtained from each patient.

### Cell lines and culture conditions

The human gastric cancer cell lines MKN28, MKN45, HGC27, MGC803, BGC823, AGS, and human immortalized gastric epithelial cells GES1 were purchased from the Institute of Biochemistry and Cell Biology, Chinese Academy of Sciences, Shanghai, China. All the cells were cultured in RPMI 1640 medium (Invitrogen, Carlsbad, CA, USA) supplemented with 10% fetal bovine serum (FBS) and 1% penicillin/streptomycin (P/S).The cells were incubated in a humidified atmosphere at 37 °C containing 5% CO_2_.

### Lentiviral transfection

Genomeditech (Shanghai, China) assisted in the design and production of CLDN6 shRNA. Gastric cancer cells were seeded in 6-well plates and incubated until cells were approximately 50% confluent. Depending on the MOI value of gastric cancer cells, appropriate lentivirus was added to each well. The cells were screened using antibiotics recommended by the company, and the transfection efficiency was measured by fluorescence quantitative PCR or western blotting. The target sequences of CLDN6 were sh-1, sense: 5' GGGATTGTCTTTGTCATCTCA 3 ‘; sh-2, sense: 5' GAGTACCCTACCAAGAATTAC 3'; sh-control, sense: 5' TTCTCCGAACGTGTCACGT 3'.

### RNA extraction and quantitative real-time PCR

Total RNA was extracted using Trizol and reverse transcribed into cDNA by PrimeScriptTM (Takara Biomedical Technology, Beijing). Using 18S rRNA as an internal control, real-time PCR analysis was performed on the 7500 real-time PCR system (Applied Biosystems), and the relative expression levels of target genes were calculated by the −ΔΔCt method. The primer sequences used are listed in Supplementary Table S1.

### Western blotting

MKN28 and AGS gastric cancer cells were lysed using RIPA lysis buffer (Beyotime, Beijing, China) and protease inhibitors (Roche, CA, USA). The proteins were separated by 10% SDS polyacrylamide gel electrophoresis and subsequently transferred onto nitrocellulose (NC) membranes. The membranes were blocked in TBS buffer containing 5% skim milk for 1 h at room temperature. After incubation with primary and secondary antibodies, ECL reagent was used to visualize the protein bands. CLDN6 (ab107059, Abcam), p-LATS1/2 (ab111344, Abcam), LATS1/2 (202761-AP, Proteintech), p-YAP1 (ab76252, Abcam), YAP1 (13584-1-AP 6900-1-Ig, Proteintech), snail1 (13099-1-AP, Proteintech), twist1(25465-1-AP, Proteintech), zeb1 (21544-1-AP, Proteintech), E-cadherin (20874-1-AP, Proteintech), N-cadherin (GB111009, Servicebio), Vimentin (GB11192, Servicebio), Ki67 (GB13030, Servicebio), β-actin (GB11001, Servicebio), HRP (GB23301, GB23303, Servicebio), Lamin B1 (AB0054, Abways).

### Coimmunoprecipitation (Co-IP)

Total protein extraction buffer (Beyotime, China) was used to extract proteins from MKN28 and AGS gastric cancer cells. Protein A/G magnetic beads (B23201, Bimake, China, Shanghai) were pre-incubated with anti-YAP1 or anti-snail1 antibody on a spinning wheel at 4 °C for 30–60 min and washed 3 times. The antibody complex was then added to the protein solution. After the protein solution was fully combined with the magnetic bead–antibody complex, the complex was washed thrice with extraction buffer. The immunoprecipitate was collected by magnetic bead separation, heated, and western blotting was performed.

### ChIP assay

ChIP assay was performed using the Pierce Agarose ChIP Kit (Thermo, 26156). Antibodies against snail1 (1:50, #3879, Cell Signaling Technology) was used for IP. The primers used for the ChIP assay are listed in Supplementary Table S2. RNA was extracted from the immunoprecipitate and reverse transcribed into cDNA for PCR analysis. RNA enrichment was measured by RT-PCR using primers specific for CDH1.

### Cell proliferation assay

Proliferation capacity of gastric cancer cells was determined by the Cell Counting Kit 8 assay (CCK-8, Beyotime, China). After transfection with CLDN6 shRNA, gastric cancer cells were inoculated into 96-well plates (approximately 2000 cells per well). Cells were incubated with 10 µl of CCK-8 reagent for 1 h in the dark, and optical density was measured at a wavelength of 450 nm using SpectrumMax Plus384 microplate reader (Molecular Devices). MKN28 and AGS gastric cancer cells (approximately 500 cells/well) were seeded in 6-well plates, and cultured for approximately 2 weeks. After washing twice in PBS, cells were fixed with 4% paraformaldehyde for 15 min, and staining was performed by adding 0.5% crystal violet for 15 min. The number of gastric cancer cell clones in different groups was calculated.

### Wound healing assay

Approximately 5 × 10^5^ cells were added to each well of a 6-well plate and cultured until completely confluent on the second day. Straight lines were drawn vertically on the monolayer with a sterile pipette tip. The cells were then washed with PBS for 3 times, and serum-free medium was added. Photographs were taken at 0, 6, 12, and 24 h.

### Transwell assay

Transwell chamber was prepared, 600 µl serum-free RPMI 1640 medium was added into the upper chamber, and 5 × 10^5^ cells were seeded in each well. The culture medium containing 20% serum was added in the lower chamber. After 24 h, the chamber was washed twice, 4% paraformaldehyde was used to fix the cells for 15 min, and crystal violet was added for 15 min to stain the cells. The number of cells passing through the chamber was counted under the microscope.

### Immunohistochemistry and staining evaluation

Immunohistochemistry (IHC) was performed as previously reported^17^. Paraffin sections were dewaxed using xylene, rehydrated by fractional ethanol, and antigens were extracted. The sections were blocked with 10% bovine serum albumin (BSA), incubated with primary antibody for 1 h, and then incubated with secondary antibody for 30 min at room temperature. DAPI (4, 6-diamino-2-phenylindole hydrochloride; AppliChem, A4099) was used to stain the nucleus. Automated fluorescence microscope (Nikon) was used for imaging and analysis. The tissue sections were assessed and graded by two independent investigators who were unaware of the clinicopathological factors. The staining intensity scoring was 0 (negative), 1 (weak), 2 (medium), and 3 (strong).The degree of staining was stratified as 0 (0%), 1(1–25%), 2 (26–50%), 3 (51–75%), 4 (76–100%), and defined as the percentage of positive staining area in the total tumor invasion area. The final score of CLDN6 expression was approximately 0–7. The samples were divided into two groups: low CLDN6 expression (0–3 points) and high CLDN6 expression (4–7 points).

### Cellular immunofluorescence

After immersion of the slides in 100% alcohol, residual alcohol was removed by flaming and ultraviolet irradiation. The glass slides were placed in a 6-well plate, and gastric cancer cells were added onto the glass slide, allowing the cells to grow up to 30–50% of the slide area on the second day. Cells were fixed with 4% paraformaldehyde for 15 min and treated with 0.5% Triton X-100 for 1 min. Non-specific binding sites were blocked by 1% BSA. The cells were incubated with the primary antibody for 1 h at room temperature, with the secondary antibody for 30 min, and treated with DAPI for 30 min for nuclear staining. The cells were observed and analyzed using an automated fluorescence microscope (Nikon).

### Animal models

To generate the subcutaneous tumor model in nude mice, 5 × 10^6^ MKN18 cells were injected into the left axilla of each nude mouse. After 4 weeks, the nude mice were sacrificed, the subcutaneous tumor weight and volume were measured, and 4% paraformaldehyde was used to fix the tumor tissue. To generate the liver metastasis model, nude mice were anesthetized with 0.5% pentobarbital, the abdominal cavity was opened, and 1 × 10^6^ BGC823 cells were injected into the spleen. After 4 weeks, the nude mice were sacrificed and liver metastases were observed. All tissues were fixed with 4% paraformaldehyde. All animal experiments were approved by the Ethics Committee of Renji Hospital.

### Statistical analysis

The SPSS 22.0 software (SPSS Inc., Chicago, IL, USA) was used to analyze the data and mean ± SD was calculated. Student’s *t* test and chi-square test were used in the study. Cox proportional hazard model was used for univariate and multivariate analyses to understand the factors affecting survival. Results with *p* < 0.05 were considered as statistically significant.

## Results

### CLDN6 is upregulated in gastric cancer

**Fig. 1:**
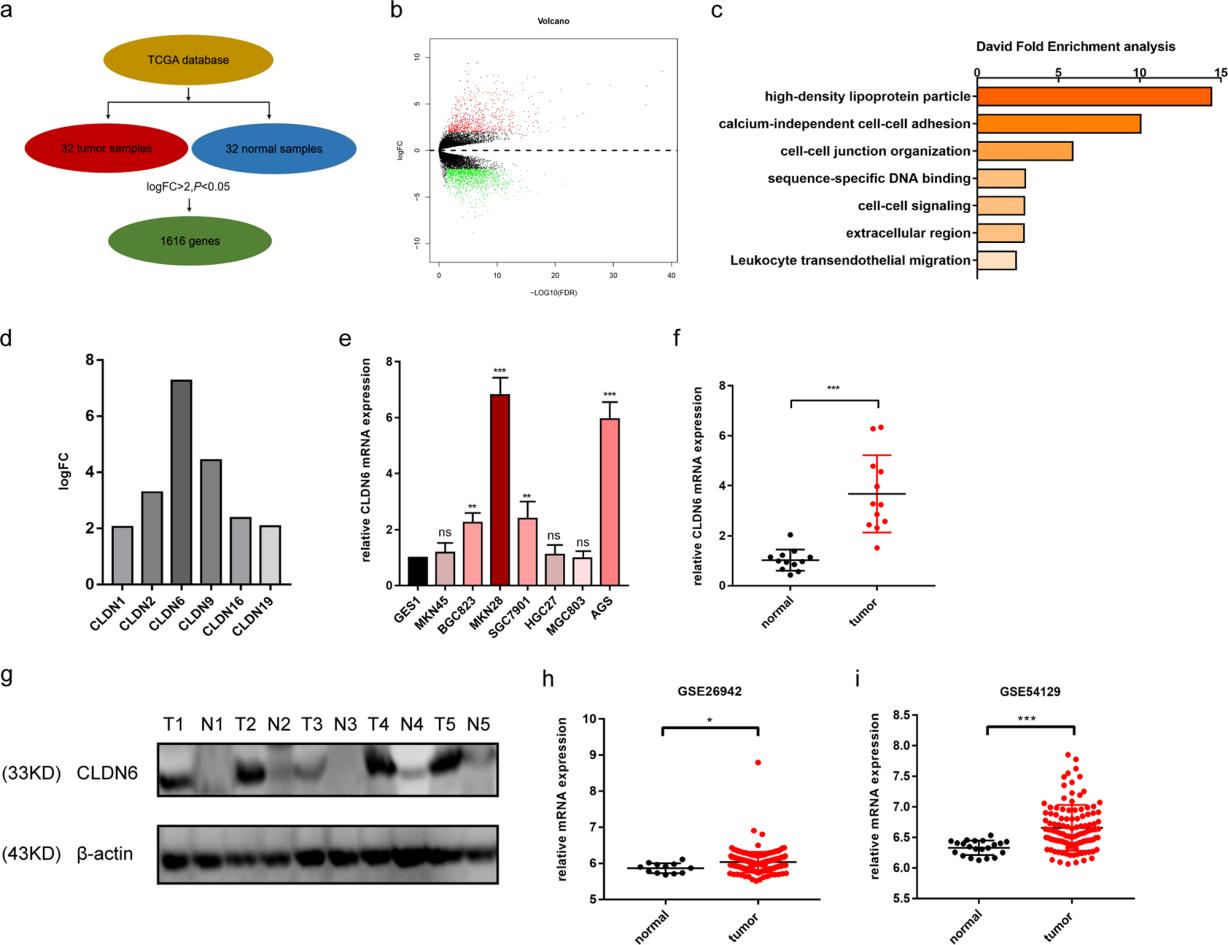
CLDN6 is upregulated in gastric cancer. **a** Screening process of differentially expressed genes from TCGA dataset (tumor samples [*n* = 32], normal samples [*n* = 32]). **b** Upregulated and downregulated genes in volcano plot. **c** Major pathways identified from DAVID fold enrichment analysis. **d** Expression profiles of different claudins whose logFC > 2. **e** Relative CLDN6 mRNA expression in eight different gastric cell lines. **f** Relative CLDN6 mRNA expression in gastric tissues (*n* = 16 in each group, student’s *t* test). **g** Relative CLDN6 protein expression in gastric tissues. **h, i**. Relative CLDN6 mRNA expression in GSE26942 and GSE54129 datasets (student’s *t* test). Each experiment was performed three times and measurement data were presented as the mean ± SD. ns, non-significant; *p < 0.05, **p < 0.01; ***p < 0.001.

Thirty-two pairs of matched gastric tissues from the TCGA dataset were selected for bioinformatics analysis. Differentially expressed genes were identified with the criteria set as *p* < 0.05 and logFC > 2 (Fig. 1a).These genes were divided into upregulated and downregulated groups (Fig. 1b). Pathway analysis using the DAVID tool revealed genes that were closely related to cell–cell adhesion and cell junction (Fig. 1c). CLDN1, 2, 6, 9, 16, and 19 were found to be of significance in gastric cancer in the tight junction family, and CLDN6 expression was the highest (Fig. 1d). Subsequently, through fluorescence quantitative PCR, we found that CLDN6 was highly expressed in MKN28 cell line with increased malignancy in GC (Fig. 1e). CLDN6 expression in gastric cancer tissues was also significantly higher than adjacent normal tissues (Fig. 1f, g), and showed a similar pattern in the GEO datasets GSE26942 and GSE54129 (Fig. 1h, i). Therefore, these results suggested that CLDN6 played a pro-tumorigenic role in gastric cancer.

### Increased CLDN6 expression is associated with poor prognosis in GC patients

**Fig. 2:**
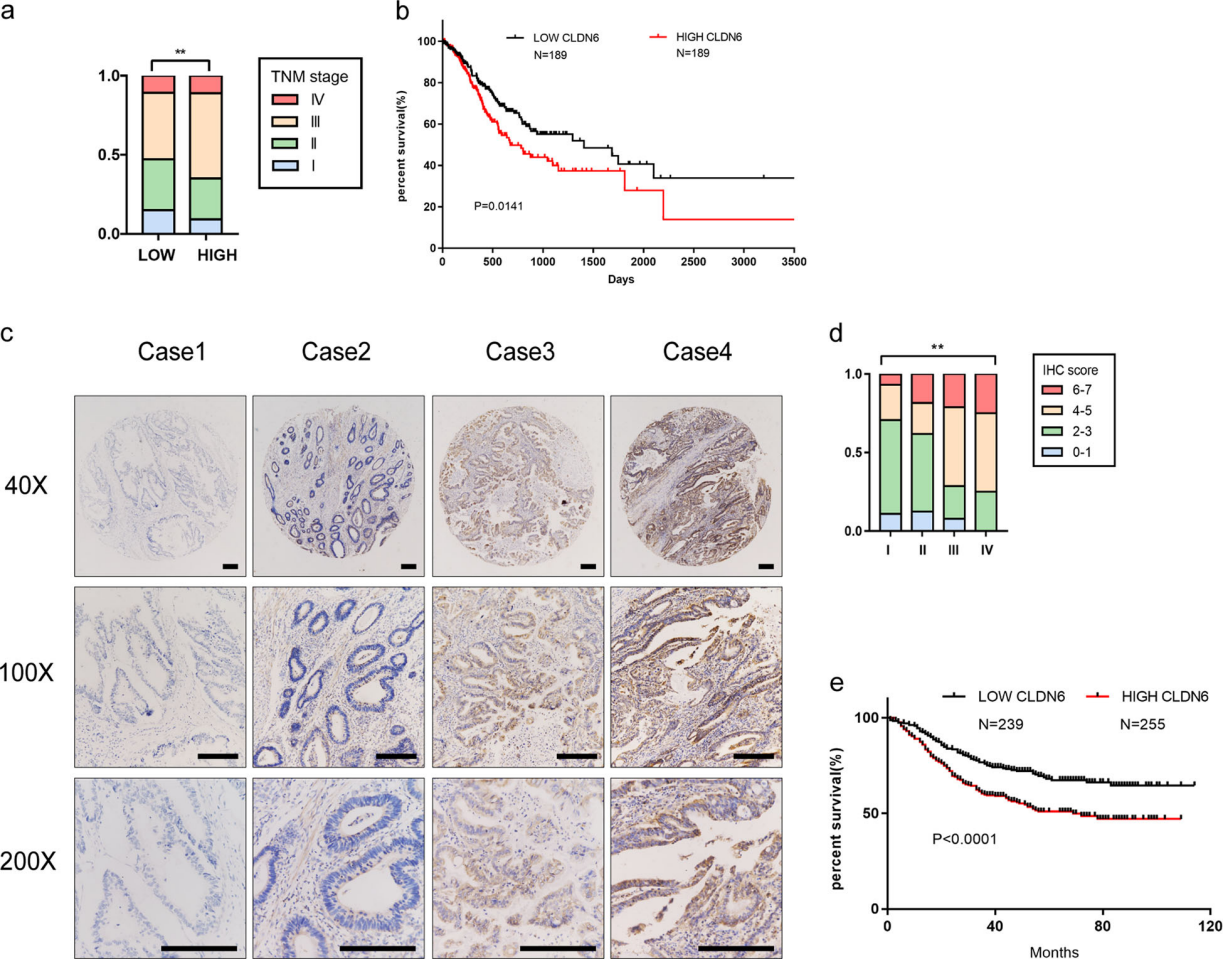
Increased CLDN6 expression is associated with worse prognosis in GC patients. **a** Proportion of different TNM stage in low CLDN6 and high CLDN6 expression groups (Spearman’s rank correlation). **b** Overall survival analysis of TCGA dataset by Kaplan-Meier Plotter (TCGA database: low CLDN6 group *n* = 189, high CLDN6 group *n* = 189). **c**. CLDN6 protein expression in GC from tissue microarray (scale bar = 100 µm). **d** Different IHC score of different pathological stages in GC (Spearman’s rank correlation). **e**. Overall survival analysis of tissue microarray by Kaplan–Meier Plotter (GC tissue microarray: low CLDN6 group *n* = 239, high CLDN6 group *n* = 255). Each experiment was performed three times and measurement data were presented as the mean ± SD. ns, non-significant; *p < 0.05;**p < 0.01; ***p < 0.001.

To investigate if CLDN6 could predict the survival of gastric cancer patients, we analyzed the data from GEO and TCGA databases as well as from Renji hospital. In TCGA database, when the patients were divided into two groups based on CLDN6 expression, we found that the number of patients with advanced-stage disease was more in high CLDN6 expression group (Fig. 2a). We also observed that the survival curve of GC patients with high CLDN6 expression indicated poor prognosis (Fig. 2b). Immunohistochemical staining for CLDN6 expression was performed on tissue microarray of 494 gastric cancer samples from Renji hospital (Fig. 2c). We found that the proportion of high staining score of CLDN6 increased gradually with TNM stage (Fig. 2d). The survival curve showed that patients with high expression of CLDN6 had worse prognosis (Fig. 2e). Moreover, we found that high CLDN6 expression was associated with age (>62 years), tumor diameter, vessel carcinoma embolus, T stage, N stage and lymph node metastasis in GC patients (Table 1). Furthermore, univariate analysis revealed that tumor diameter, tumor embolus, nerve invasion, T stage, N stage, lymph node involvement, and high CLDN6 expression were risk factors in GC, and multivariate analysis showed that tumor embolus and T stage were the major risk factors (Supplementary Table S3).

**Table 1:** The relationship between CLDN6 expression and clinical factors.

		CLDN6 expression		
Parameters	*n* = 494	Low (*n* = 239)	High (*n* = 255)	*p*-value
*Age*				0.012
≤62	247	134	113	
>62	247	105	142	
*Gender*				0.558
Male	342	162	180	
Female	152	77	75	
*Tumor diameter*				<0.001
≤4 cm	255	143	112	
>4 cm	239	96	143	
*Tumor embolus*				<0.001
No	419	220	199	
Yes	75	19	56	
*Nerve invasion*				0.215
No	435	215	220	
Yes	59	24	35	
*N stage*				<0.001
N0	192	144	48	
N1-N3	302	95	207	
*T stage*				<0.001
T0-T2	147	92	55	
T3-T4	347	147	200	
*Node involvement*				<0.001
No	197	145	52	
Yes	297	94	203	

Pearson χ^2^ test

### CLDN6 promotes proliferation and invasion abilities of GC in vitro

**Fig. 3:**
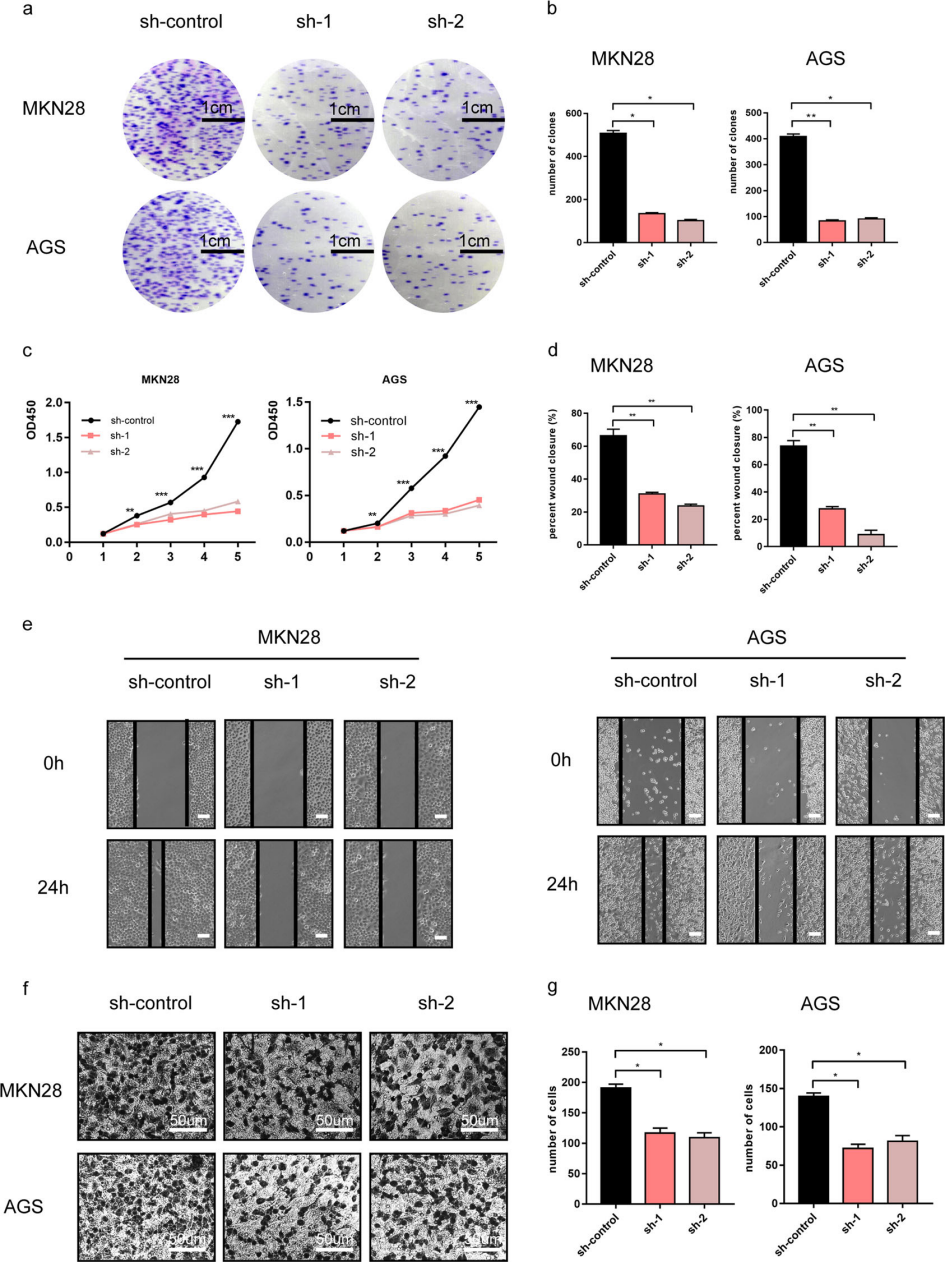
CLDN6 promotes proliferation and invasion abilities of GC in vitro. **a, b** Colony formation assays showed the effects of CLDN6 knockdown in MKN28 and AGS gastric cancer cell growth (student’s *t* test, scale bar = 1 cm). **c** Cell viability of MKN28 and AGS was measured by CCK8 assay (student’s *t* test). **d, e** Wound healing assay showing the effect of silencing CLDN6 expression in MKN28 and AGS cells (student’s *t* test, scale bar = 100 µm). **f, g** Transwell assay showing the migration ability of MKN28 and AGS cells after knockdown of CLDN6 expression (student’s *t* test, scale bar = 50 µm). Each experiment was performed three times and measurement data was presented as the mean ± SD. ns, non-significant; *p < 0.05;**p < 0.01; ***p < 0.001.

To further understand the biological function of CLDN6 in gastric cancer, shRNA was used to knockdown CLDN6 expression in MKN28 and AGS cells, and the knockdown efficiency was confirmed by fluorescence quantitative PCR (Supplementary Fig. S1). The colony formation and CCK8 assays revealed that the proliferative ability of gastric cancer cells was inhibited after suppression of CLDN6 expression (Fig. 3a-c). Wound healing and transwell assays showed that the migration ability of gastric cancer cells significantly decreased upon CLDN6 knockdown (Fig. 3d-g). Together, these results suggest that silencing CLDN6 expression inhibited the proliferation and invasion abilities of GC in vitro.

### CLDN6 promotes proliferation and invasion abilities in GC in vivo

**Fig. 4:**
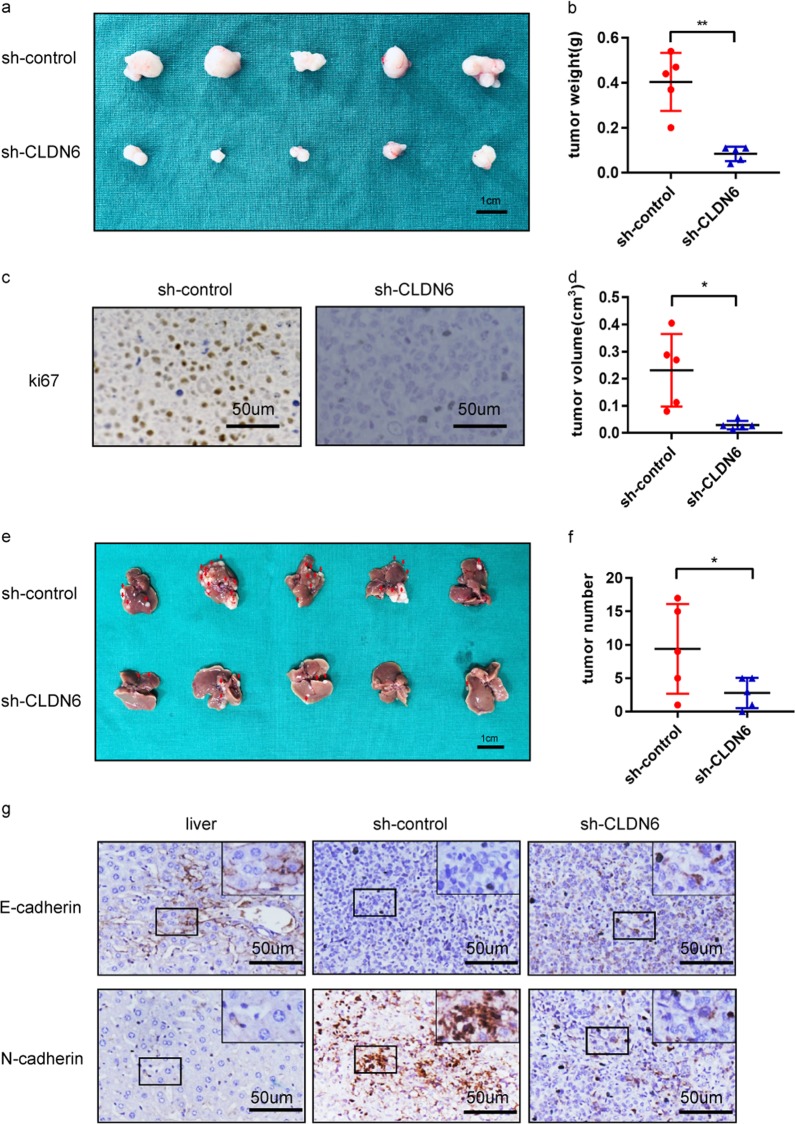
CLDN6 promotes proliferation and invasion abilities of GC in vivo. **a** Nude mice were injected with sh-CLDN6 or sh-control vector transfected cells (sh-CLDN6 [*n* = 5] or sh-control [*n* = 5], scale bar = 1 cm). **b** Tumor weight of sh-control and sh-CLDN6 groups (student’s *t* test). **c** Subcutaneous tumors were stained with Ki67 in sh-CLDN6 and sh-control group (scale bar = 50 µm). **d** Tumor volume of sh-control and sh-CLDN6 groups (student’s *t* test). **e** Tumor growth assessment in liver metastasis model of GC (red arrows indicate metastasis, scale bar = 1 cm). **f** Tumor numbers in sh-control and sh-CLDN6 groups in liver metastasis model of GC (*n* = 5 in each group, student’s *t* test). **g** E-cadherin and N-cadherin staining of adjacent normal tissues (liver tissue group) and tumor tissues (sh-CLDN6 and sh-control groups, scale bar = 50 µm). Each experiment was performed three times and measurement data was presented as the mean ± SD. ns, non-significant; *p < 0.05;**p < 0.01; ***p < 0.001.

To evaluate whether abnormal expression of CLDN6 enhanced tumorigenicity of gastric cancer in vivo, gastric cancer cells transfected with sh-CLDN6 and empty vector constructs, were injected subcutaneously and into the spleen of nude mice, respectively, to generate xenograft subcutaneous tumor model and liver metastasis model (Fig. 4a, e). The weight and volume of the transplanted tumors from nude mice were measured and the number of liver metastases from the liver metastasis model was calculated. The subcutaneous tumor weight and volume were significantly reduced in the sh-CLDN6 group (Fig. 4 a, b, d). Moreover, the sh-control group was prone to liver metastasis of gastric cancer, because the number of liver metastases in this group increased significantly (Fig. 4 e, f). Immunohistochemical staining of subcutaneous tumors showed that Ki67 expression in sh-CLDN6 group was significantly lower than sh-control group (Fig. 4c). In addition, strong E-cadherin and weak N-cadherin staining was observed in sh-CLDN6 group (Fig. 4g). Collectively, these results indicated that high CLDN6 expression enhanced the proliferative and invasive abilities of tumors in vivo, which was consistent with our previous in vitro results.

### CLDN6 promotes YAP1 nuclear translocation that interacts with snail1 to promote EMT

**Fig. 5:**
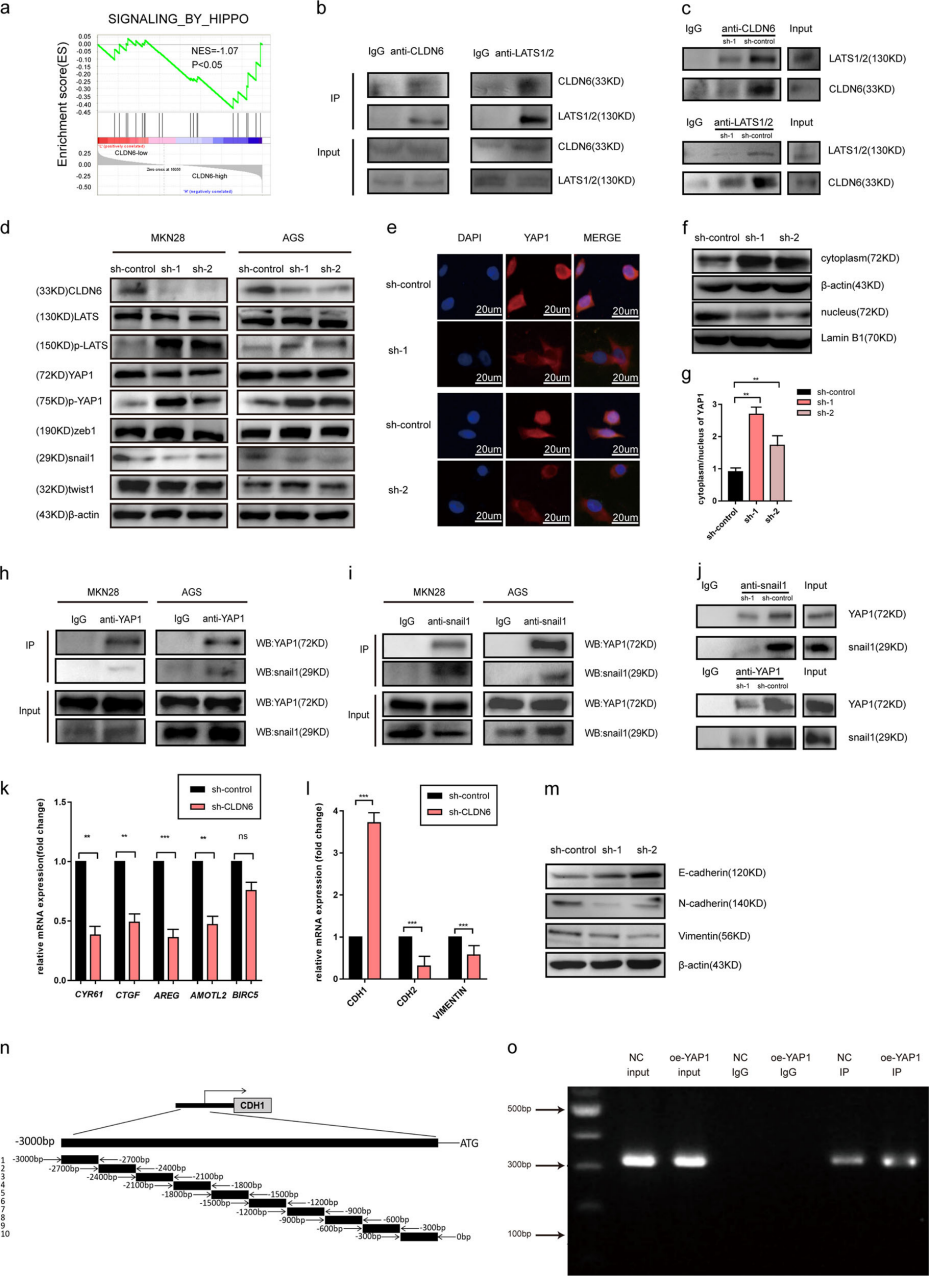
CLDN6 promotes YAP1 nuclear translocation that interacts with snail1 to promote the process of EMT. **a** GSEA analysis was performed for CLDN6 expression in GC, including low CLDN6 expression group (40 samples) and high CLDN6 expression group (40 samples) from GSE110875. **b, c** Immunoprecipitation assay showed the interaction between CLDN6 and LATS1/2. **d** Protein levels of CLDN6, LATS, p-LATS, YAP1, p-YAP1, zeb1, snail1, twist1, and β-actin were measured in MKN28 and AGS cells by western blotting. **e** Cellular immunofluorescence assay showed YAP1 localization in MKN28 GC cells in sh-control group and sh-CLDN6 group (scale bar = 20 µm). **f, g** Nuclear extraction experiment showed the quantification of the cytoplasm/nucleus of YAP1. **h, i, j** Immunoprecipitation assay showed the interaction between YAP1 and snail1 in MKN28 and AGS cells. **k** mRNA levels of downstream genes of YAP1 in sh-control and sh-CLDN6 groups by RT-PCR. **l** mRNA levels of CDH1, CDH2, and Vimentin in sh-control and sh-CLDN6 groups by RT-PCR. **m** Protein levels of E-cadherin, N-cadherin, and Vimentin in sh-control and sh-CLDN6 groups by western blotting. **n** Schematic of primer design for CDH1 promoter sequences. Ten primer sets with a 300-bp partition were designed for PCR to test the direct binding of YAP1-snail1 to the CDH1 promoter and the primer pairs produced 10 fragments of 300 bp. **o** Amplification of CDH1 promoter sequence from ChIP DNA validated the binding of YAP1-snail1 to the CDH1 promoter site. Every experiment was performed three times and measurement data was presented as the mean ± SD. ns, non-significant; *p < 0.05;**p < 0.01; ***p < 0.001.

Our in vitro and in vivo experimental results revealed that the abnormal expression of CLDN6 was related to the proliferation and invasion abilities of gastric cancer. Therefore, we explored the possible mechanism of CLDN6 in promoting the progression of gastric cancer. The GSE110875 dataset contained 80 gastric cancer samples, which were divided into low and high CLDN6 expression groups. We then used GSEA to analyze the changes in related pathways, and identified the Hippo signaling pathway to be of significance (Fig. 5a). The immunoprecipitation assay results showed that CLDN6 and LATS1/2 interacted with each other (Fig. 5b), thus causing phosphorylation and dephosphorylation changes in downstream YAP1. This interaction became weak after we silenced CLDN6 expression (Fig. 5c). In MKN28 and AGS cell lines, western blot assay showed that p-LATS and p-YAP1 levels were increased significantly in the sh-CLDN6 group (Fig. 5d).The increased levels of p-YAP1 led to decreased YAP1 entry into the nucleus, thereby inhibiting the development of gastric cancer (Fig. 5e). We also observed through cellular immunofluorescence assay and nuclear extraction experiment that, compared to the sh-CLDN6 group, sh-control group showed significantly increased expression of YAP1 in the nucleus(Fig. 5e–g) and activated the expression of downstream target genes such as *CYR61*, *CTGF*, *AREG*, and *AMOTL2* (Fig. 5k). Because our previous experiments showed that reduced CLDN6 expression affected the invasion ability of gastric cancer cells, we speculated that CLDN6 was closely associated with the process of EMT. Western blotting results showed that knockdown of CLDN6 expression caused significant changes in snail1 protein, while there were no significant alterations in zeb1 and twist1 (Fig. 5d). Therefore, we reasonably speculated that CLDN6 might promote EMT through the YAP1-snail1 axis. Interestingly, the coimmunoprecipitation assay confirmed the interaction between YAP1 and snail1 (Fig. 5 h, i) and, YAP1 did not interact with either twist1 or zeb1 (Supplementary Fig. S3) in MKN28 and AGS cells. The interaction between YAP1 and snail1 became weak after we silenced CLDN6 expression (Fig. 5j). GC cells were transfected with sh-CLDN6, and protein and mRNA levels of EMT markers were evaluated. Results showed that the expression level of E-cadherin significantly increased, while the levels of N-cadherin and vimentin significantly decreased in sh-CLDN6 group (Fig. 5 l, m). Subsequently, ChIP assay was performed and we observed that the YAP1-snail1 complex indeed bound to the promoter of CDH1 at approximately −300 bp and, when we overexpressed YAP1, the expression was stronger than before (Fig. 5 n, o).These results indicated that CLDN6 activated the expression of related oncogenes by increasing YAP1 nuclear translocation, thus accelerating the proliferation of GC cells. In addition, the interaction between YAP1 and snail1 promoted EMT of gastric cancer cells, thereby promoting invasion of gastric cancer.

### Overexpression of YAP1 partially rescued the diminished proliferation and invasion abilities caused by CLDN6 knockdown

**Fig. 6:**
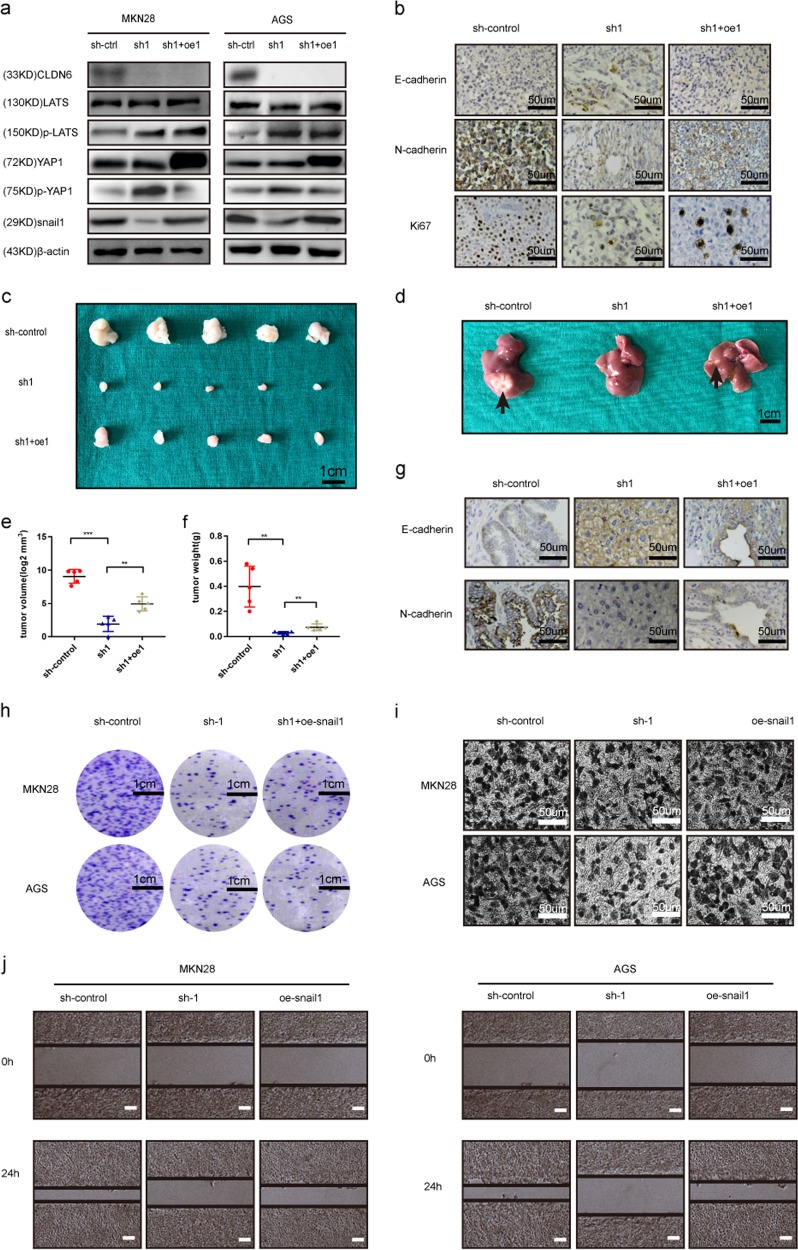
Overexpression of YAP1 partially rescued the diminished proliferative and invasive ability caused by silencing CLDN6 expression. **a** Protein levels of CLDN6, LATS, p-LATS, YAP1, p-YAP1, snail1, and β-actin were measured in MKN28 and AGS cells by western blotting in sh-control, sh-CLDN6 and sh-CDLN6 + oe-YAP1S127A group. **b** Subcutaneous tumors in each group were stained with E-cadherin, N-cadherin and Ki67 (scale bar = 50 µm). **c** Nude mice were injected with sh-control vector, sh-CLDN6 and sh-CDLN6 + oe-YAP1S127A transfected cells (sh-control [*n* = 5], sh-CLDN6 group [*n* = 5], sh-CDLN6 + oe-YAP1S127A [*n* = 5], scale bar = 1 cm). **d** Tumor growth assessment in liver metastasis model of GC in different groups (*n* = 3 in each group, black arrows show metastasis, scale bar = 1 cm). **e**, **f** Tumor volume and weight of sh-control, sh-CLDN6, and sh-CDLN6 + oe-YAP1S127A groups (student’s *t* test). **g** Liver metastatic tissues were stained for E-cadherin or N-cadherin (sh-CLDN6 group had no liver metastasis, scale bar = 50 µm). **h** Colony formation assay showed the effects of snail1 overexpression on MKN28 and AGS gastric cancer cell growth (student’s *t* test, scale bar = 1 cm). **i** Transwell assay showed the migration ability of MKN28 and AGS cells after snail1 overexpression (student’s *t* test, scale bar = 50 µm). **j** Wound healing assay showed the influence of snail1 overexpression on MKN28 and AGS cells (student’s *t* test, scale bar = 100 µm). Every experiment was performed three times and measurement data was presented as the mean ± SD. ns, non-significant; *p < 0.05; **p < 0.01; ***p < 0.001.

To further understand the CLDN6-YAP1-snail1 axis, we overexpressed YAP1S127A (one of the phosphorylation sites of YAP1 was mutated) in MKN28 and AGS cells that were transfected with sh-CLDN6 previously. Western blot analysis showed that after the overexpression of YAP1 in sh-CLDN6 group, the expression of LATS1/2 upstream remained unchanged, while the expression of snail1 was relatively increased downstream (Fig. 6a). Overexpression of YAP1 partially rescued the effect of diminished expression of CLDN6 on snail1. Subcutaneous tumor model showed that suppression of CLDN6 expression reduced tumor load, while overexpression of YAP1 reversed this trend of tumor load reduction (Fig. 6 c, e and f). In addition, in the liver metastasis model, liver tissues were more likely to have metastasis in the sh-CLDN6 + oe-YAP1 group compared to the sh-CLDN6 group (Fig. 6d). Moreover, immunohistochemical staining was performed to evaluate the expression of Ki67, E-cadherin, and N-cadherin. We found that overexpression of YAP1 partially reversed the tumor-inhibiting effect CLDN6 knockdown. (Fig. 6 b, g).To further evaluate the effect of CLDN6-YAP1-snail1 axis, we again overexpressed snail1 in sh-CLDN6 GC cells. We found that snail1 overexpression partially increased the cell invasion ability but not the proliferation ability (Fig. 6 h-j). Based on these results, we speculated that YAP1 was able to moderately rescue the diminished effects caused by CLDN6 knockdown and CLDN6-YAP1-snail1 axis affected the proliferation and invasion abilities of gastric cancer.

## Discussion

Claudin proteins play critical roles in human tumors, affecting cell proliferation, differentiation, migration, metastasis, apoptosis, and other processes^18–21^. For instance, claudin18 was found to regulate lung stem cell and progenitor cell heterogeneity, and tumor formation by affecting YAP activity^22^. A previous study also demonstrated that claudin18 could regulate the proliferation of pulmonary epithelial cells^23^. Another study revealed that downregulation of claudin3 could highly activate the Wnt/β-catenin and IL6/gp130/Stat3 signaling pathways, and enhance the malignancy of colorectal cancer^24^. It has been suggested that claudin2 on the surface of tumor cells could interact with liver cells to promote liver metastasis of breast cancer^25^. These studies largely reflected the role of claudin proteins in the process of tumorigenicity by activating or inactivating downstream signaling pathways, which were closely related to epithelial cell homeostasis, invasion, chronic inflammation, and cancer. Claudin proteins could also promote EMT through zeb-1/E-cadherin, Wnt signaling, MMP9/Notch signaling, and other signaling pathways^26^. In this study, we found that claudin 6 is highly expressed in gastric cancer, especially in a cell and tissue type specific manner with high malignancy potential. The abnormal expression of claudin6 resulted in EMT, indicating poor prognosis. Further, through GSEA analysis, we found that the aberrant expression of claudin6 was closely associated with the Hippo signaling pathway. The upstream membrane protein of the Hippo signaling pathway senses the extracellular growth inhibition signal and activates a kinase cascade resulting in a series of phosphorylation reactions, and finally phosphorylates the downstream effectors YAP1 and TAZ^27–29^. Cytoskeletal proteins bind to phosphorylated YAP1 and TAZ, retaining them in the cytoplasm and thereby reducing their nuclear activity^30–32^. Essentially, YAP1 is associated with the process of EMT. There have been studies reporting that the interaction between KRAS and YAP1 could regulate EMT and tumor survival^33^. In addition, the self-renewal of liver cells was dependent on YAP1-TGF-β-induced EMT^34^. It has been reported that the *Helicobacter pylori* CagA protein promoted the process of EMT in gastric cancer by triggering the tumorigenic YAP pathway^35^. In this study, we found that claudin6 reduced the phosphorylation level of downstream YAP1 by interacting with LATS1/2, thereby increasing the nuclear activity of YAP1. Subsequently, immunoprecipitation assay showed that YAP1 interacted with snail1 and influenced the expression of downstream target genes such as *CDH1*, *CDH2*, and *Vimentin*, thereby causing EMT in gastric cancer. Claudins play an important role in cancer development and the corresponding claudin monoclonal antibodies designed to treat cancer are expected to be highly specific, which help in reduce the side effects of chemotherapy and thereby improve treatment efficacy. Currently, monoclonal antibodies have been produced for CLDN1, CLDN2, CLDN3, CLDN4, CLDN6, and CLDN18.2^26^. IMAB027, a monoclonal antibody against CLDN6, has been tested in phase I/II trials for ovarian cancer^36^. If the monoclonal antibody therapy shows promise, efforts are required to extrapolate this strategy to treat gastric cancer.

**Fig. 7:**
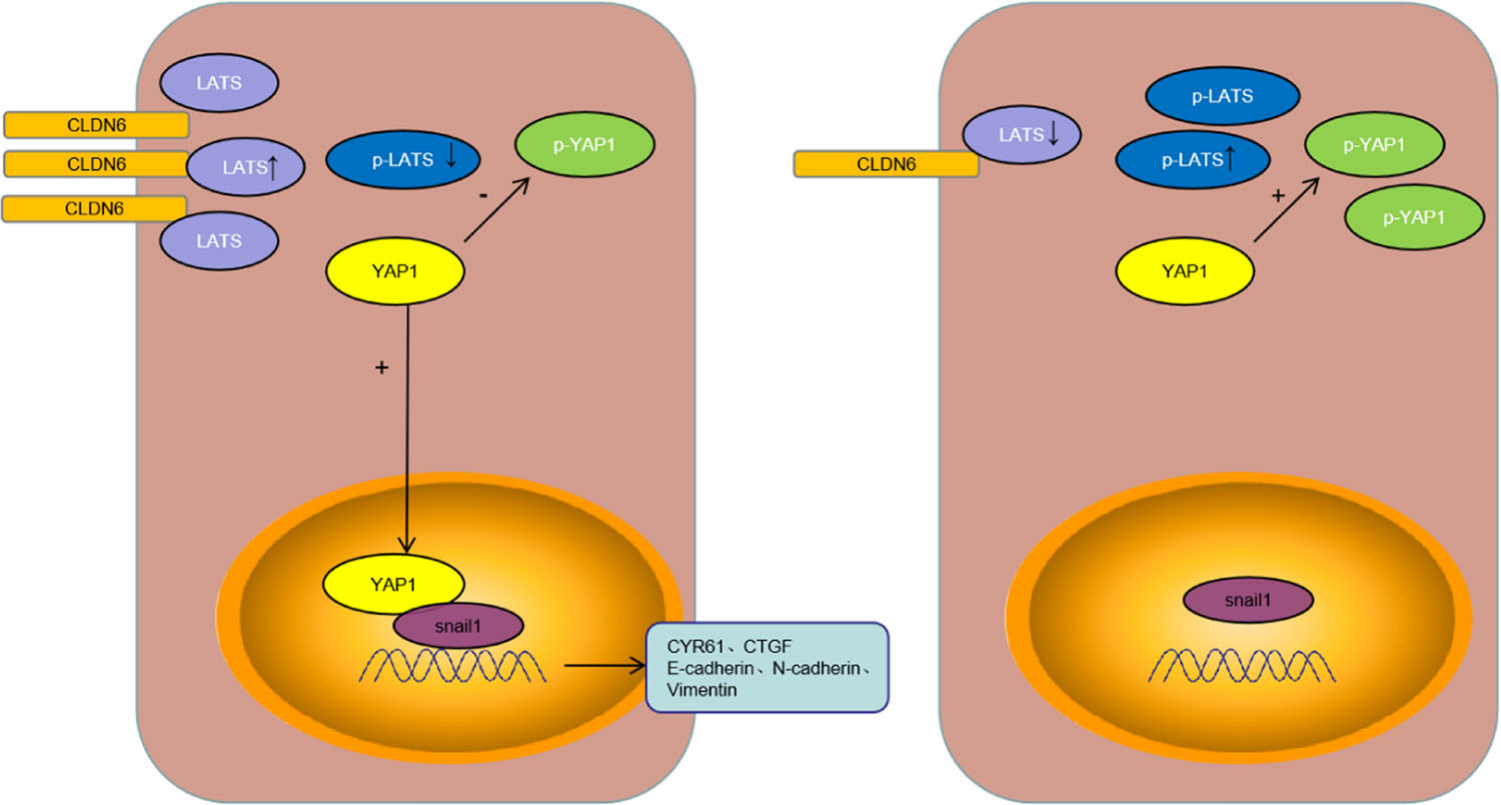
Schematic representation of the mechanism of CLDN6-YAP1-snail1 axis. Upregulated CLDN6 expression: CLDN6 interacted with LATS, reducing the conversion of LATS into p-LATS, and YAP1 into p-YAP1. Therefore, increased amount of YAP1 entered cell nucleus to activate its downstream target genes. Increased YAP1 interacted with snail1 to bind to the promoter of EMT related genes to enhance EMT progression. Downregulated CLDN6 expression: Decreased amount of CLDN6 interacted with LATS thereby increasing p-LATS and resulting in increased p-YAP1. Due to decreased levels of YAP1 in the cell nucleus, the downstream pathways were inhibited.

There are still certain limitations in this study. First, in the animal model of liver metastasis, we found that the nude mice in sh-CLDN6 group were less prone to liver metastasis than the sh-control group. So far, we have little understanding of the mechanism by which CLDN6 promotes liver metastasis in GC. As reported in a previous study, claudins on tumor cells interacted with claudins in liver cells, promoting cancer cell growth in liver tissues. The process of liver metastasis involves a complex mechanism, which requires further exploration. Second, although the immunoprecipitation assay results showed that CLDN6 interacted with LATS1/2, the way their interaction affected the downstream proteins is unclear. We have tested the protein levels of LATS, p-LATS, YAP1, and p-YAP1 by western blot analysis. We speculated that the interaction of LATS1/2 with CLDN6 relatively decreased the conversion of LATS into p-LATS, thereby inhibiting the Hippo signaling pathway. Upon CLDN6 knockdown, the interaction between CLDN6 and LATS was diminished, which led to an increase in p-LATS thus activating the Hippo signaling pathway (Fig. 7). However, an in-depth study needs to be carried out to elucidate these mechanisms.

In summary, the present study found the pro-tumorigenic effect of CLDN6 in gastric cancer and revealed that CLDN6 promoted YAP1 nuclear translocation by reducing YAP1 phosphorylation, thereby activating downstream oncogenes. It promoted EMT of gastric cancer through YAP1-snail1 interaction, thus enhancing the proliferation and invasion abilities of gastric cancer cells. This study provides new insights to design relevant therapeutic strategies for the treatment of gastric cancer.

## Supplementary information

Table S1

Table S2

Table S3

Supplemetary Figure 1

Supplemetary Figure 2

Supplemetary Figure 3

Supplemetary Figure 3
