# Supplementary material for: CLDN6 promotes tumor progression through the YAP1-snail1 axis in gastric cancer
Source: Cell Death Dis. 2019 Dec 11;10(12):949. doi: 10.1038/s41419-019-2168-y (PMC6906326; doi:10.1038/s41419-019-2168-y)
Supplement: Supplementary file 1 — Table S1 [file 41419_2019_2168_MOESM1_ESM.doc]

| Gene symbol | Forward or reverse | sequence (5' -> 3') |
| --- | --- | --- |
| CLDN6 | Forward Primer | TGTTCGGCTTGCTGGTCTAC |
|  | Reverse primer | CGGGGATTAGCGTCAGGAC |
| CDH1 | Forward Primer | CGAGAGCTACACGTTCACGG |
|  | Reverse primer | GGGTGTCGAGGGAAAAATAGG |
| CDH2 | Forward Primer | AGCCAACCTTAACTGAGGAGT |
|  | Reverse primer | GGCAAGTTGATTGGAGGGATG |
| Vimentin | Forward Primer | AGTCCACTGAGTACCGGAGAC |
|  | Reverse primer | CATTTCACGCATCTGGCGTTC |
| CYP61 | Forward Primer | ACCGCTCTGAAGGGGATCT |
|  | Reverse primer | ACTGATGTTTACAGTTGGGCTG |
| CTGF | Forward Primer | CAGCATGGACGTTCGTCTG |
|  | Reverse primer | AACCACGGTTTGGTCCTTGG |
| AREG | Forward Primer | GTGGTGCTGTCGCTCTTGATA |
|  | Reverse primer | CCCCAGAAAATGGTTCACGCT |
| AMOTL2 | Forward Primer | CTCAGCGTGACACCACTCTC |
|  | Reverse primer | GTGAGCAGACCCTCATTGAAG |
| BIRC5 | Forward Primer | AGGACCACCGCATCTCTACAT |
|  | Reverse primer | AAGTCTGGCTCGTTCTCAGTG |
| RPS18 | Forward Primer | ATCACCATTATGCAGAATCCACG |
|  | Reverse primer | GACCTGGCTGTATTTTCCATCC |

Table S1 Primer used in the assays.
