# Supplementary material for: CLDN6 promotes tumor progression through the YAP1-snail1 axis in gastric cancer
Source: Cell Death Dis. 2019 Dec 11;10(12):949. doi: 10.1038/s41419-019-2168-y (PMC6906326; doi:10.1038/s41419-019-2168-y)
Supplement: Supplementary file 2 — Table S2 [file 41419_2019_2168_MOESM2_ESM.doc]

| CDH1 | Forward or reverse | sequence (5' -> 3') |
| --- | --- | --- |
| 1 | Forward Primer | AAATTGAGGGGACAGGTTGTTGGGG |
|  | Reverse primer | CTGTGACCTTGGGGAAGTCAGTGTT |
| 2 | Forward Primer | GGCTAGTGAGTGGCTGACTCCACTT |
|  | Reverse primer | TCACCTTCTCCAGAGAATTCTGTCC |
| 3 | Forward Primer | TGGTGGCCATGGTGTGGCTGTGGCA |
|  | Reverse primer | AGATTGAGGCCAATGAGAGGTGGCG |
| 4 | Forward Primer | CTCTAAGCTTCAGTTGCCCATCTAT |
|  | Reverse primer | CTCTTGGGTTCAAGCGGTTCTCCTC |
| 5 | Forward Primer | GCGAAGGTTGCAGTGAGCCAAGAAC |
|  | Reverse primer | TGTAGTCCCAGCTACTCGGGAGGCT |
| 6 | Forward Primer | GGCGCCCACCACCACGCCTGGCTAA |
|  | Reverse primer | AGTTTCTCCACCCTCCTAATGGGAA |
| 7 | Forward Primer | GAGGCTTTGGGAGGTGGTCCTGACC |
|  | Reverse primer | TGGTCTGGAACTCCTGACCTCAGGT |
| 8 | Forward Primer | GCCTGGCCAACATGGTGAAACCCCG |
|  | Reverse primer | ATCCTCCTGCCTTGGCCTCCCAAAG |
| 9 | Forward Primer | CGCTTCAGCCCAGGAGTTCGAGACC |
|  | Reverse primer | TTCTTTTGTTTTTTGGGATTTTTTG |
| 10 | Forward Primer | CTCAGCCAAGTGTAAAAGCCCTTTC |
|  | Reverse primer | GAGAGGGGGTGCGTGGCTGCAGCCA |

Table S2 CDH1 Primer used in the ChIP assay.
