# Supplementary material for: CLDN6 promotes tumor progression through the YAP1-snail1 axis in gastric cancer
Source: Cell Death Dis. 2019 Dec 11;10(12):949. doi: 10.1038/s41419-019-2168-y (PMC6906326; doi:10.1038/s41419-019-2168-y)
Supplement: Supplementary file 3 — Table S3 [file 41419_2019_2168_MOESM3_ESM.docx]

|  |  | univariate |  |  |  | multivariate |  |
| --- | --- | --- | --- | --- | --- | --- | --- |
| variables | HR | 95%CI | P value |  | HR | 95%CI | P value |
| Age(≤62） | 0.819 | 0.621-1.080 | 0.819 |  |  |  |  |
| Gender(male) | 0.886 | 0.661-1.188 | 0.419 |  |  |  |  |
| tumor diameter(≤4cm) | 0.398 | 0.297-0.532 | <0.001 |  | 0.771 | 0.561-1.060 | 0.110 |
| tumor embolus（no） | 0.316 | 0.230-0.434 | <0.001 |  | 0.548 | 0.387-0.777 | 0.001 |
| Nerve invasion(no) | 0.421 | 0.295-0.601 | <0.001 |  | 0.738 | 0.505-1.080 | 0.118 |
| T stage(T0-T2) | 0.221 | 0.142-0.344 | <0.001 |  | 0.414 | 0.253-0.676 | <0.001 |
| N stage(N0) | 0.224 | 0.154-0.325 | <0.001 |  | 0.504 | 0.119-2.142 | 0.353 |
| Node involvement(no) | 0.229 | 0.159-0.330 | <0.001 |  | 0.713 | 0.175-2.915 | 0.638 |
| low CLDN6 expression | 0.543 | 0.408-0.722 | <0.001 |  | 1.031 | 0.751-1.414 | 0.851 |

Table S2 Univariate and multivariate analysis for predictors of overall survival in microarray of GC.
