# Supplementary material for: CLDN6 promotes tumor progression through the YAP1-snail1 axis in gastric cancer
Source: Cell Death Dis. 2019 Dec 11;10(12):949. doi: 10.1038/s41419-019-2168-y (PMC6906326; doi:10.1038/s41419-019-2168-y)
Supplement: Supplementary file 4 — Supplemetary Figure 1 [file 41419_2019_2168_MOESM4_ESM.docx]

**Supplementary Figure Legends**

**Supplementary Figure 1.** Heatmap showing the profile of differentially expressed genes of 32 paired samples from TCGA dataset.

**Supplementary Figure 2.** Knockdown efficiency of CLDN6 expression upon transfection with sh-CLDN6 in MKN28 and AGS GC cells.

**Supplementary Figure 3.** **a.** Immunoprecipitation assay showed no interaction between YAP1 and zeb1 in MKN28 cells. **b.** Immunoprecipitation assay showed no interaction between YAP1 and twist1 in MKN28 cells.
