# Supplementary figures and images for: CLDN6 promotes tumor progression through the YAP1-snail1 axis in gastric cancer
Source: Cell Death Dis. 2019 Dec 11;10(12):949. doi: 10.1038/s41419-019-2168-y (PMC6906326; doi:10.1038/s41419-019-2168-y)

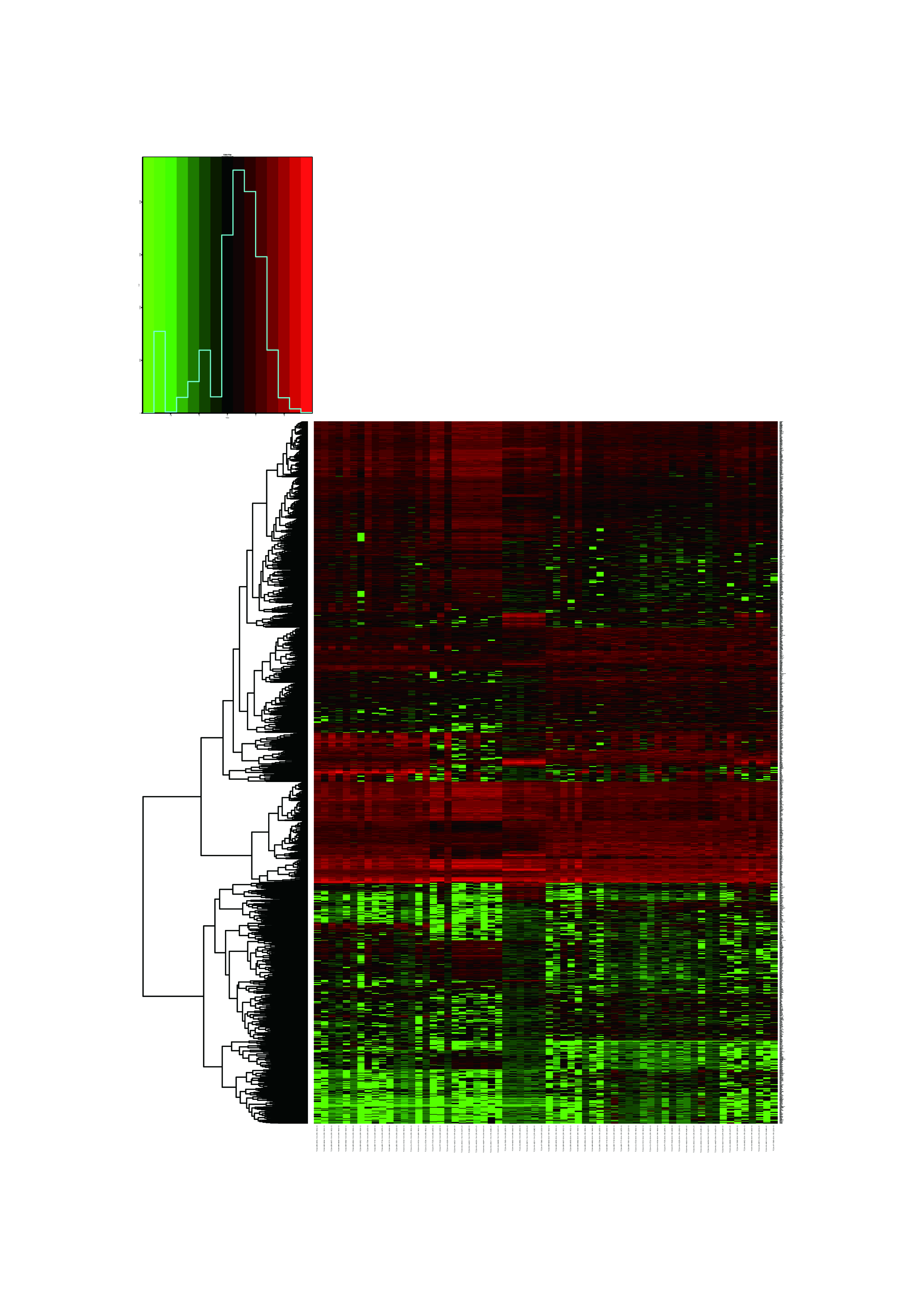

Supplement: Supplementary file 5 — Supplemetary Figure 2 [file 41419_2019_2168_MOESM5_ESM.tif]

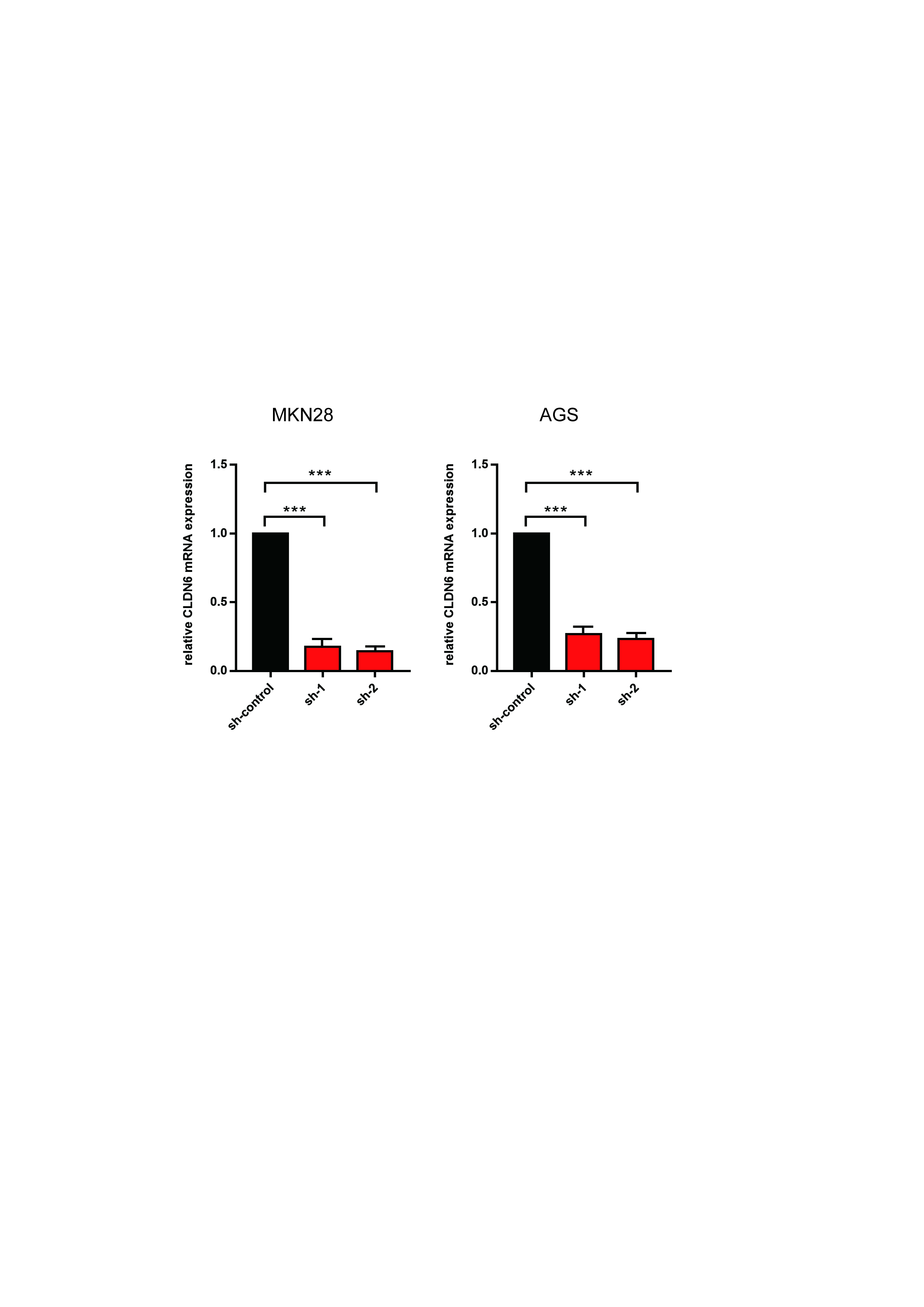

Supplement: Supplementary file 6 — Supplemetary Figure 3 [file 41419_2019_2168_MOESM6_ESM.tif]

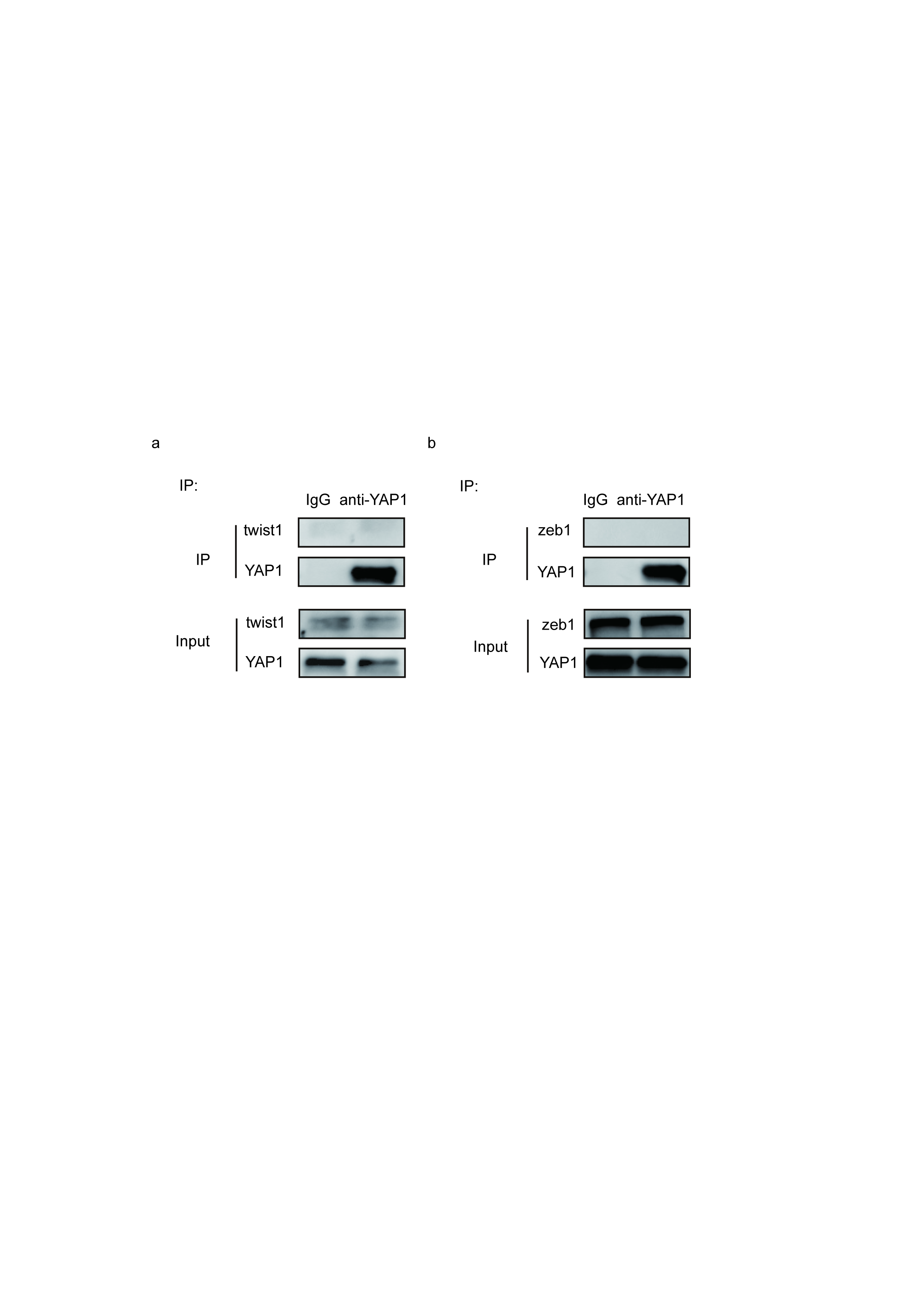

Supplement: Supplementary file 7 — Supplemetary Figure 3 [file 41419_2019_2168_MOESM7_ESM.tif]
